# Great Expectations in Durham

**Published:** 1920-02-28

**Authors:** 


### Great Expectations in Durham.

The woman who helps in a working-class household is expected to do a miscellany of things; and, to ensure that the new corps of home-helps under the maternity and child welfare scheme shall know precisely what to do, the Durham County Council's Health Committee has compiled a list of those things which should be done and also of those things which must be left undone.

Thus, the helps must undertake cooking, working, and house cleaning, but not whitewashing or paperhanging. The fact that the County Council finds it necessary to schedule whitewashing and paperhanging as prohibited occupations seems to suggest that some families expect their houses to be renovated and put in first-class order when the municipal help comes along. Home-helps are forbidden to undertake nursing, except for attention necessary between visits of midwife or district nurse. Times of duty are from 8 a.m. to 6 p.m., six days a week. Wages are 20s. weekly if food is provided, and 26s. without food. Helps may undertake duties on odd days at a wage of 3s. with food, or 4s. without food.

